# Geovisualizing land degradation risk in Southeast Brazil Using remote sensing and GIS-based assessment

**DOI:** 10.1038/s41598-026-49578-w

**Published:** 2026-07-15

**Authors:** Mohammad AlAbed, Turkia Almoustafa, Roberson Pimentel, Fábio F. Dias

**Affiliations:** 1 Department of Geoenvironmental Analysis, Fluminense Federal University (UFF), Niterói, Brazil; 2 Department of Geography, University of Manchester, Manchester, UK; 3 Department of Animal Science and Sustainable Agro-Socio-environmental Development, Fluminense Federal University (UFF), Niterói, Brazil

**Keywords:** Spatial multi-criteria analysis, Soil erosion, Conservation prioritization, Land use transition, Atlantic forest biome, Climate sciences, Ecology, Ecology, Environmental sciences, Environmental social sciences, Geography, Geography, Natural hazards

## Abstract

Land degradation poses a critical threat to ecosystem services and sustainable development, especially in regions experiencing rapid land-use transitions. This study employs an integrated geospatial approach—combining remote sensing, geographic information systems (GIS), and spatial multi-criteria analysis—to assess and visualize land degradation risk in a strategic coastal region of southeastern Brazil (Rio de Janeiro State). Using the United Nations Environment Programme’s Priority Actions Programme Regional Activity Centre (UNEP-PAP/RAC) framework applied to recent satellite imagery, we generated spatially explicit maps classifying land into stable and unstable categories. A geospatial prioritization model incorporating biophysical and socio-economic variables was developed to identify conservation hotspots and support decision-making. Results show that 68.4% of the landscape is stable, largely consisting of unmanaged areas with agricultural and forest potential, while 7.8% is unstable, with sheet erosion concentrated at agricultural frontiers. Priority mapping classified 51.7% of the area as Stable Medium Priority, revealing widespread latent vulnerability, and 4.6% as Unstable High Priority, necessitating urgent intervention. Complementary analysis of land-use change (1985–2024) highlighted a 199% urban expansion and a 34% decline in agricultural mosaics, underscoring anthropogenic drivers of degradation. This study not only validates the PAP/RAC framework in a humid tropical coastal setting but also delivers actionable geovisualization outputs and a spatial decision-support tool for targeted land management, contributing to soil conservation and sustainable development policy in Brazil.

## Introduction

Land degradation represents a critical environmental and socio-economic challenge, threatening ecosystem services, agricultural productivity, and sustainable development worldwide^1^. In Brazil, a country of vast natural resources and complex biomes, soil erosion driven by anthropogenic activities such as agricultural expansion, deforestation, and urbanization is a persistent issue, particularly in regions undergoing rapid land-use transition^2^. The state of Rio de Janeiro, while known for its coastal metropolises, encompasses diverse hinterlands characterized by mosaics of Atlantic Forest remnants, agricultural lands, and urban-rural interfaces. These areas are susceptible to degradation processes, yet spatially explicit assessments integrating modern geospatial technologies remain essential for informing targeted conservation and land-use policies.

**Fig. 1:**
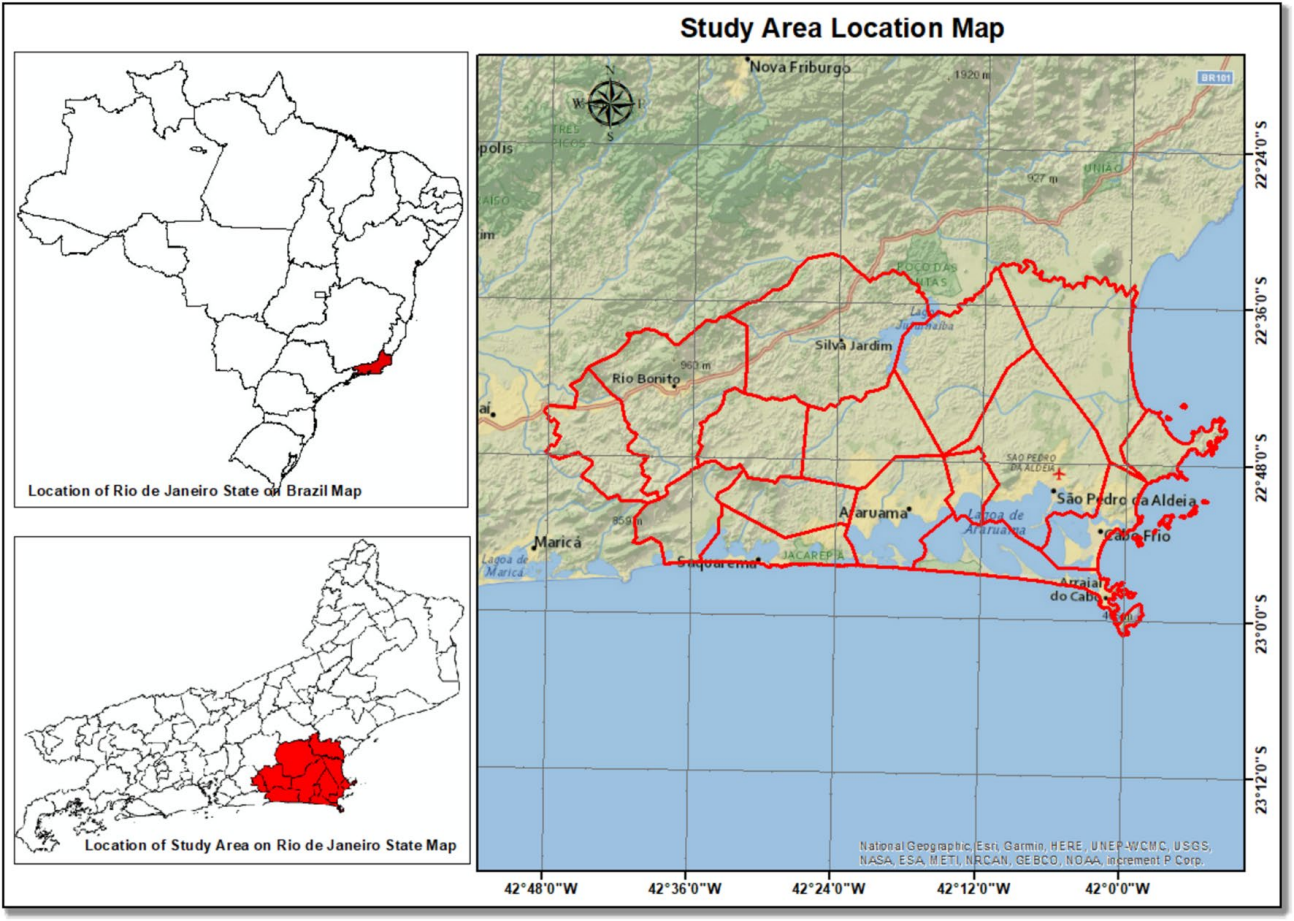
Location map of the study area. Map created using ArcGIS version 10.8 (https://www.esri.com/en-us/arcgis/products/arcgis-desktop/resources).

The current study focuses on a strategically selected area in the southeast of Brazil. As shown in the location map (Fig. 1), the study area is positioned in the eastern part of the state of Rio de Janeiro, spanning approximately 3020 km2 between the coordinates 42°48’0"W to 42°30’0"W and 23°00’0"S to 22°30’0"S. This area represents a quintessential land-use transition zone, featuring interfaces between the Atlantic Forest biome, cultivated lands, coastal systems (including mangroves and sandy areas), and expanding urban peripheries. Such heterogeneity makes it a pertinent model for studying the spatial patterns and drivers of land degradation.

This study adopts three interrelated concepts that structure the analytical framework. *Land stability* refers to the capacity of a land unit to maintain its structure, function, and productive potential under prevailing biophysical and anthropogenic pressures. *Stable Area (Non-Erosion Affected Area)* refers to an area of land with no evidence of active erosion processes, indicating a state of morphodynamic equilibrium due to the stabilizing effect of landscape components. *Unstable Area (Erosion Affected Area)* refers to an area of land where one or several active erosion processes occur, such as sheet or rill erosion. *Conservation priority* is defined as the relative urgency for intervention, determined by integrating biophysical vulnerability with socio-economic factors that influence the feasibility and necessity of management actions^3^.

Remote sensing (RS) and Geographic Information Systems (GIS) have emerged as indispensable tools for mapping, monitoring, and modeling land degradation at various scales^4^. The integration of these technologies enables the systematic analysis of land cover change, identification of erosion features, and assessment of degradation risk over large and often inaccessible areas. The UNEP-PAP/RAC framework offers a structured, multi-criteria approach that is globally applicable for assessing land degradation risk and prioritizing interventions. Originally developed for Mediterranean coastal zones, its utility extends to diverse environmental settings worldwide, including humid tropical regions, drylands, and mountainous areas where anthropogenic pressures drive soil erosion. By integrating biophysical stability assessments with socio-economic prioritization, the framework supports evidence-based land management decisions that align with international sustainability goals such as Land Degradation Neutrality (LDN) and SDG 15.3 (Life on Land). Adapting and validating such a framework in contrasting environmental contexts is essential for developing globally robust tools to combat land degradation. However, the application of integrated RS/GIS frameworks for detailed risk assessment, particularly those that classify land into stability categories and conservation priorities, remains underexplored for many dynamic regions in southeastern Brazil. While integrated RS/GIS land degradation assessments are well-established globally, three specific gaps motivate this study. First, the UNEP-PAP/RAC framework—despite its structured approach to classifying degradation types and intervention priorities—has been predominantly applied in semi-arid and Mediterranean contexts (e.g.,^5–8^. Its application in humid tropical coastal zones characterized by heavy rainfall, steep relief, and complex land-use mosaics addresses a significant methodological gap^9^. Second, existing assessments often separate biophysical mapping from socio-economic prioritization,this study integrates both through a multi-criteria framework that explicitly links stability classification to intervention urgency. Third, by combining degradation mapping with 40-year LULC change analysis, this study establishes empirical relationships between land-use transitions and degradation hotspots—a dimension often absent from static assessments.

The main objectives of this research are to: (1) map and quantify the spatial distribution of stable and unstable land classes; (2) analyze the associated erosion processes; (3) develop a conservation priority map by synthesizing stability status and degradation risk with biophysical and socio-economic variables; and (4) establish empirical linkages between land-use/land-cover change (1985–2024) and observed degradation patterns. The findings are intended to provide a scientific basis for land management planning, supporting strategies for soil conservation, ecosystem restoration, and sustainable regional development in one of Brazil’s most environmentally and economically significant states.

## Material and methods

### Land degradation mapping

Land degradation was mapped using a contemporary, very-high-resolution remote sensing approach. The primary data sources consisted of the most recent available very-high-resolution (<1 m) satellite basemaps to ensure fine-scale accuracy: Airbus imagery (2025) and the Esri World Imagery service. Land cover classification was performed in ArcGIS using the Maximum Likelihood Supervised Classification algorithm. The classification schema was designed according to the established methodological framework of the United Nations Environment Programme’s Priority Actions Programme/Regional Activity Centre (UNEP-PAP/RAC) for coastal area management^3^^,^^10^. Training samples for the supervised classification were developed and iteratively refined through visual interpretation of the very-high-resolution Airbus and Esri basemaps. A total of 1,248 training polygons were delineated, comprising approximately 41,802 pixels distributed across all land cover classes defined by the UNEP-PAP/RAC framework. Sample sizes were determined to ensure a minimum of 50 pixels per class, required for robust covariance matrix estimation, with classes exhibiting higher spectral heterogeneity allocated proportionally larger samples. This visual refinement against sub-meter imagery was critical for defining precise spectral signatures and ensuring high classification accuracy in the absence of concurrent field validation.

The classification procedure followed a dual-track logic based on land unit status, distinguishing between stable and unstable areas. Stable land units were defined and characterized based on three primary attributes: (1) the dominant land cover/use type (e.g., sandy areas, unmanaged areas with forest or agricultural potential, managed forest or agricultural areas, mangrove); (2) an inherent instability risk index (0 = no risk, 1 = low, 2 = high, 3 = highest), derived from intrinsic factors; and (3) the primary drivers of potential instability, including topography, geology, vegetation cover, and anthropogenic pressure. Unstable land units were identified and classified based on: (1) the dominant erosion process (sheet, or rill erosion); (2) the spatial extent of the affected area within the unit (localized: <30%, dominant: 30–60%, widespread: >60%); and (3) a qualitative expansion trend index indicating the dynamic state of the erosion (0 = stabilizing, 1 = locally expanding, 2 = regionally expanding, 3 = advancing toward irreversibility).

### Prioritization of degradation hotspots

To facilitate efficient resource allocation and targeted intervention, a systematic prioritization procedure was applied to identify critical land degradation hotspots. This procedure was developed following the methodological framework established by the United Nations Environment Programme’s Priority Actions Programme/Regional Activity Centre^11^. Fourteen variables were selected based on their direct relevance to local land degradation processes and prevailing socio-economic dynamics (Table 1). Variables were categorized into actual degradation risk factors (A–E), socio-economic factors (F–I), and land use factors (J–N).

**Table 1:** Variables and scoring matrix for land degradation intervention prioritization.

Variable	Description	Scoring (1–3)
*A*	Physical instability risk (stable areas)	1 = low, 2 = high, 3 = critical
*B*	Extent of area affected (unstable areas)	1 = <30%, 2 = 30–60%, 3 = >60%
*C*	Expansion trend of degradation (unstable areas)	1 = local, 2 = widespread, 3 = generalized/irreversible
*D*	Multiplicator for increased importance (causative agents or degradation process)	1 = none, 2 = increased, 3 = highly increased
*E*	Influence on adjacent areas	1 = low, 2 = high, 3 = critical
*F*	Overexploitation	1 = insignificant, 2 = significant, 3 = crucial
*G*	Rural exodus	
*H*	Land tenure	
*I*	Other aggravating socio-economic factors	
*J*	Value of current land use (local population)	1 = low, 2 = moderate, 3 = high
*K*	Value of current land use (national policy)	
*L*	Potential for forestry	
*M*	Potential for agriculture	
*N*	Other land use potentials (recreational, industrial, construction)	

Each variable was assigned an impact score ranging from 1 (lowest impact) to 3 (highest impact). Actual degradation risk variables capture the intrinsic stability of land units and the severity of active degradation processes; their impact scores were taken from the land degradation map produced in section "Land degradation mapping". Variable A (physical instability risk) for stable areas was assigned directly from the stable area code in the resulting land degradation map. Variables B (extent of area affected by a specific degradation process for unstable areas) and C (expansion trend of a specific degradation process for unstable areas) were also assigned directly from the unstable area’s codes in the resulting land degradation map. Variable D (multiplicator for increased importance of causative agents for stable areas or for increased importance of a specific degradation process for unstable areas) and Variable E (influence on adjacent areas) were assessed using visual interpretation by overlapping the land degradation map with the ESRI base map to assign the increased risk importance and its influence on adjacent areas. Overexploitation (F) impact score was assessed by overlapping the land degradation map with the ESRI base map. Rural exodus (G), land tenure (H), and other aggravating socio-economic factors (I) impact scores were given the value (1) as the lowest impact because no field survey or questionnaire was conducted. Land use value variables (J–N) were scored based on visual interpretation by overlapping the land degradation map with the ESRI base map to assign the impact score (1 to 3) based on expert judgment supported by knowledge of the study area.

The final composite prioritization scores were then calculated using distinct formulas for stable and unstable areas, reflecting their different underlying risk structures:

- Stable Areas Priority Score: [(A × D + E) × F × G × H × I] + [(J + K) × L × M × N]
- Unstable Areas Priority Score: [(B × C × D + E) × F × G × H × I] + [(J + K) × L × M × N]

The multiplicative structure captures synergistic interactions where multiple high-risk factors amplify each other, while additive components allow for compensatory relationships. The resulting numerical scores were classified into three distinct intervention priority tiers to guide management actions: High Priority: Score ≥ 60, Medium Priority: Score between 21 and 59, and Low Priority: Score ≤ 20. This quantitative framework translates the multi-criteria assessment into a clear, actionable hierarchy for conservation and restoration planning.

### Spatio-temporal land use and land cover change detection (1985–2024)

**Fig. 2:**
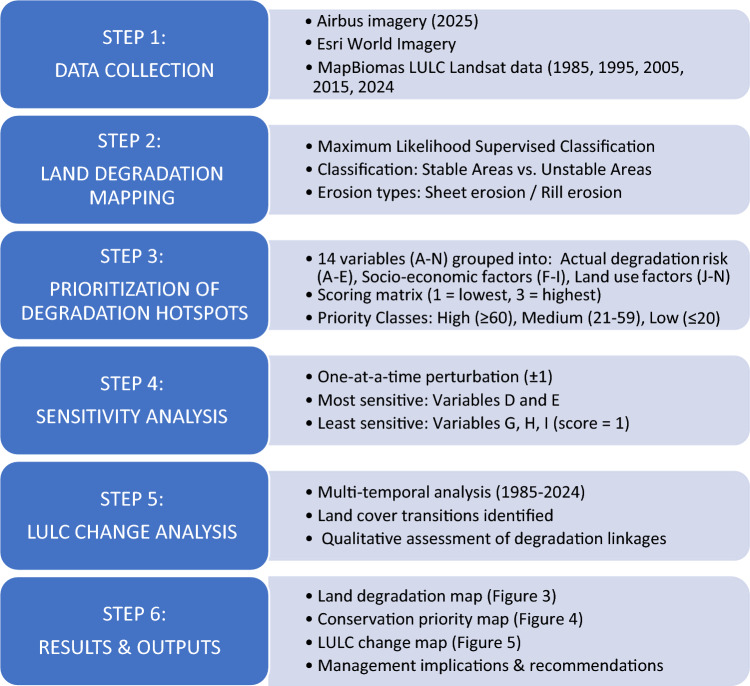
Methodological flowchart for land degradation risk assessment and conservation prioritization.

Land Use and Land Cover (LULC) data were sourced from the MapBiomas Project, a Brazilian multi-institutional initiative that generates annual, high-resolution land cover maps for Brazil and other regions using Landsat satellite imagery with a 30 m spatial resolution^12^. This study analyzed multi-temporal LULC changes at ten-year intervals (1985, 1995, 2005, 2015, and 2024). The raster datasets for these five target years were downloaded and processed in ArcGIS 10.8. Each dataset was spatially clipped to the study area’s vector boundary to isolate relevant LULC patterns. Area calculations for each land cover class were performed by aggregating pixel counts and applying the sensor’s spatial resolution, with results expressed in square kilometers, hectares, and proportional cover (%) to facilitate cross-temporal and inter-class comparison. The total study area used for all subsequent calculations is 3019.77 km^2^, derived from the official vector boundary. The initial summed area of the clipped MapBiomas raster was approximately 9% larger. This discrepancy is attributed to the inclusion of partial coastal and island pixels during the raster clipping process, a known effect when working with complex shorelines at moderate (30 m) resolution. All final class areas and percentages were calculated based on the definitive vector-based total to ensure geographical accuracy, enabling a quantitative evaluation of land cover transitions over the 40-year period. The full methodological flowchart of the integrated geospatial approach for land degradation risk assessment and conservation prioritization is presented in Fig. 2.

## Results

### Nature and extent of land degradation

**Fig. 3:**
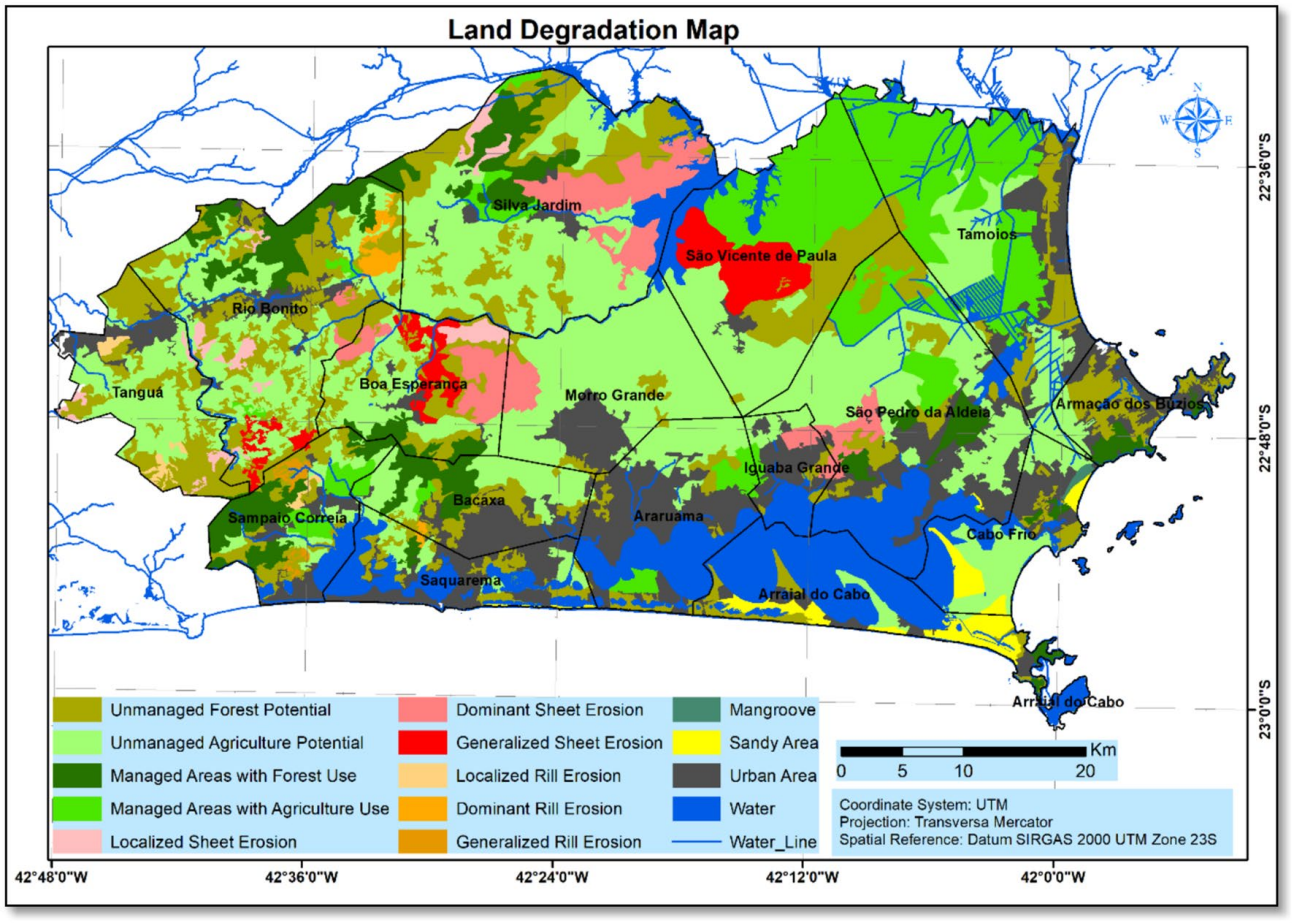
Land degradation map showing stable areas and unstable areas. Map created using ArcGIS version 10.8 (https://www.esri.com/en-us/arcgis/products/arcgis-desktop/resources).

The land degradation assessment for the study area in Rio de Janeiro State revealed a clear spatial differentiation between stable and unstable land categories, as illustrated in the land degradation map (Fig. 3) and summarized quantitatively in Table 2. The total study area covers approximately 3019.77 km^2^. Spatial analysis indicates that the landscape is predominantly characterized by stable land conditions, constituting approximately 68.4% of the total area. The most extensive stable category is Unmanaged Areas with Agriculture Potential, covering 929.33 km^2^ (30.8% of the study area). This is complemented by significant coverage of Unmanaged Areas with Forest Potential, spanning 541.29 km^2^ (17.9%). Together, these two classes form broad, contiguous land bases in municipalities such as Silva Jardim, Morro Grande, and Rio Bonito. Managed lands also contribute substantially to stable terrain: Managed Areas with Agriculture Use account for 350.45 km^2^ (11.6%), extending mainly across Tamoios and São Vicente de Paula, while Managed Areas with Forest Use cover 198.64 km^2^ (6.6%), predominantly occupying the mountainous regions of Rio Bonito and Silva Jardim. Minor yet ecologically significant stable features include Sandy Areas, occupying 38.71 km^2^ (1.3%), and Mangrove ecosystems, confined to 7.06 km^2^ (0.2%).

**Table 2:** Assessment of stable and unstable land categories.

**Areas**	**Code**	**Land category type**	**Area** **(km**^**2**^**)**	**Area** **(hec)**	**Area** **(%)**
Stable areas	0S	Sandy area	38.71	3870.67	1.28
	01	Unmanaged areas with forest potential	541.29	54128.97	17.92
	02	Unmanaged areas with agriculture potential	929.33	92933.26	30.77
	03	Managed areas with forest use	198.64	19864.48	6.58
	04	Managed areas with agriculture use	350.45	35045.13	11.61
	0M	Mangrove	7.06	706.29	0.23
	**Total stable area**		**2065.48**	**206548.8**	**68.39**
Unstable areas		Localized sheet erosion	28.25	2825.17	0.94
		Dominant sheet erosion	112.77	11277.45	3.73
		Generalized sheet erosion	71.42	7141.92	2.37
		Localized rill erosion	7.16	715.59	0.24
		Dominant rill erosion	11.06	1105.91	0.37
		Generalized rill erosion	4.79	478.61	0.16
	**Total unstable area**		**235.45**	**23544.65**	**7.81**
Urban area			411.69	41169.43	13.63
Water			307.14	30714.44	10.17
**Total surface study area**			**3019.77**	**301977.32**	**100.00**

In contrast, areas exhibiting active soil degradation, classified as unstable, constitute a smaller but notable portion of the landscape, totaling approximately 235 km^2^ of the study area. Erosion processes are dominated by sheet erosion, the most widespread form of degradation. Dominant Sheet Erosion affects 112.77 km^2^ (3.7%), followed by Generalized Sheet Erosion across 71.42 km^2^ (2.4%), and Localized Sheet Erosion over 28.25 km^2^ (0.9%). Rill erosion is less extensive but present, with Dominant Rill Erosion occurring over 11.06 km^2^ (0.4%), Localized Rill Erosion over 7.16 km^2^ (0.2%), and Generalized Rill Erosion across 4.79 km^2^ (0.2%). Spatially, unstable areas are not randomly distributed but show a distinct association with specific land use types and vulnerable geomorphological units, often occurring at the interface between managed agricultural zones and natural vegetation. The mapped unstable areas are concentrated within the central and peripheral zones of the study area, encompassing municipalities such as Rio Bonito, Boa Esperança, Silva Jardim, São Vicente de Paula, Iguaba Grande, and São Pedro. Visual assessment suggests that erosion processes are particularly associated with agricultural frontiers and areas of land-use transition. Precise quantification of erosion extent per administrative unit would require further municipal-scale spatial analysis. The remaining surface is composed of anthropogenic and permanent natural features. Urbanized areas account for 411.69 km^2^ (13.6%), while water bodies cover 307.14 km^2^ (10.2%).

### Conservation priority assessment

**Fig. 4:**
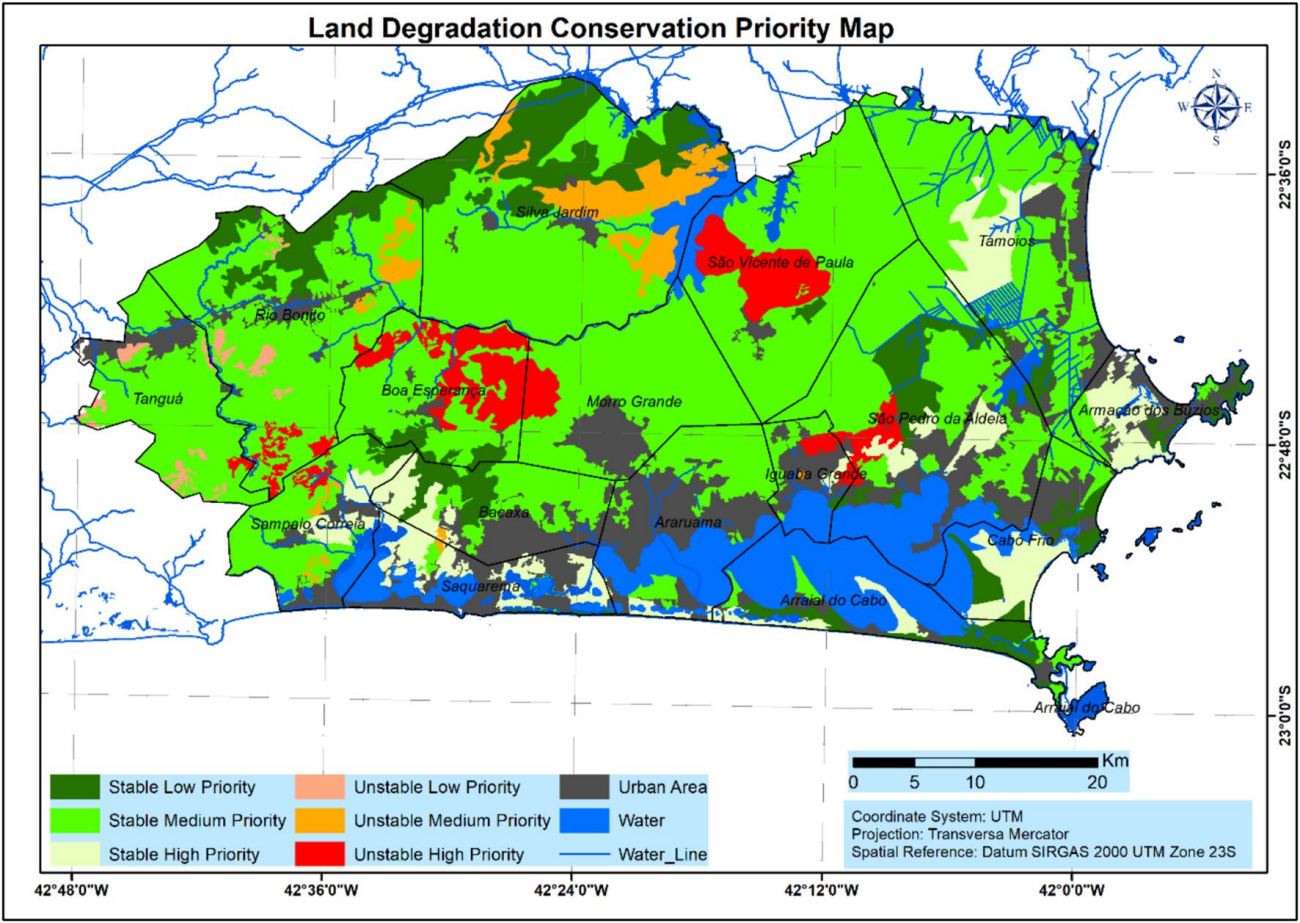
Land degradation conservation priority map. Map created using ArcGIS version 10.8 (https://www.esri.com/en-us/arcgis/products/arcgis-desktop/resources).

The land degradation assessment was extended to classify areas based on their conservation priority (Fig. 4). The resulting priority map and corresponding areal statistics (Table 3) reveal a landscape where the majority of the area is designated for medium-level conservation attention. Stable areas account for 68.37% (2064.65 km^2^) of the total study area and are subdivided into three priority levels. The largest portion is classified as Stable Medium Priority, encompassing 1560.80 km^2^ or 51.69% of the total landscape. This suggests that over half of the region, while currently stable, possesses characteristics that warrant monitoring or preventive management to maintain its condition. Stable Low Priority areas cover 309.28 km^2^ (10.24%), indicating zones of high resilience or lower degradation risk. In contrast, Stable High Priority areas, though smaller at 194.57 km^2^ (6.44%), highlight specific stable zones that are critically important for conservation, likely due to high ecological value or proximity to vulnerable features.

**Table 3:** Conservation priority assessment results.

Area	**Conservation priority**	**Area** **(km**^**2**^**)**	**Area** **(hec)**	**Area** **(%)**
stable areas	Stable low priority	309.28	30927.6	10.24
	Stable medium priority	1560.80	156080.3	51.69
	Stable HIGH PRIORITY	194.57	19456.8	6.44
	**Total stable areas**	**2064.65**	**206464.7**	**68.37**
Unstable areas	Unstable low priority	16.89	1689.16	0.56
	Unstable medium priority	77.97	7797.24	2.58
	Unstable high priority	140.17	14016.64	4.64
	**Total unstable areas**	**235.03**	**23503.04**	**7.78**
Urban area		412.32	41231.96	13.65
Water		307.58	30758.2	10.19
**Total surface area**		3019.58	301957.9	100.00

Unstable areas, representing active degradation across 7.78% (235.03 km^2^) of the study area, are further categorized by intervention urgency. The most critical category is Unstable High Priority, covering 140.17 km^2^ (4.64% of the total area). This signifies that the majority of eroded land requires immediate and targeted intervention to mitigate soil loss. Unstable Medium Priority areas span 77.97 km^2^ (2.58%), while Unstable Low Priority zones are minimal at 16.89 km^2^ (0.56%). The remaining surface is comprised of Urban Area (412.32 km^2^, 13.65%) and Water bodies (307.58 km^2^, 10.19%).

Spatially, the priority map (Fig. 4) indicates a distinct zonation. High and medium-priority unstable areas are frequently concentrated at the interface between agricultural frontiers and natural vegetation, particularly within the central and southern parts of the study area, overlapping with municipalities such as Rio Bonito, Boa Esperança, Silva Jardim, Sâo Pedro, and São Vicente de Paula. The extensive Stable Medium Priority class forms a contiguous matrix across the region, suggesting a widespread need for sustainable land use planning to prevent future degradation. This priority classification provides a strategic framework for directing conservation resources, emphasizing that preventive measures in medium-priority stable zones are as crucial as rehabilitation efforts in high-priority unstable areas.

To assess the robustness of the prioritization results to uncertainty in variable scoring, a one-at-a-time sensitivity analysis was conducted. For each variable in the composite index, scores were perturbed by ±1 (within the allowable 1–3 range), and the resulting changes in priority class assignment were evaluated. The analysis revealed that the classification was most sensitive to variables D (multiplicator for increased importance) and E (influence on adjacent areas), with modifications to these variables resulting in priority class shifts for a measurable proportion of land units. Variables J, K, L, M, and N (land use value and potential variables) also showed moderate sensitivity due to their multiplicative structure. In contrast, variables G, H, and I (rural exodus, land tenure, and other socio-economic factors) were assigned the lowest score (1) for all units because no field survey was conducted; consequently, these variables showed no sensitivity to perturbation. The Stable Medium Priority class demonstrated the greatest resilience, with only a small percentage of its area transitioning to other classes under maximum perturbation scenarios. Conversely, the boundaries between Stable Low Priority and Stable High Priority classes showed greater sensitivity, highlighting the need for refined local data in transitional zones. These results confirm the overall stability of the priority classification while identifying variables that would benefit from further validation in future applications.

### Land use and land cover change analysis (1985–2024)

**Fig. 5:**
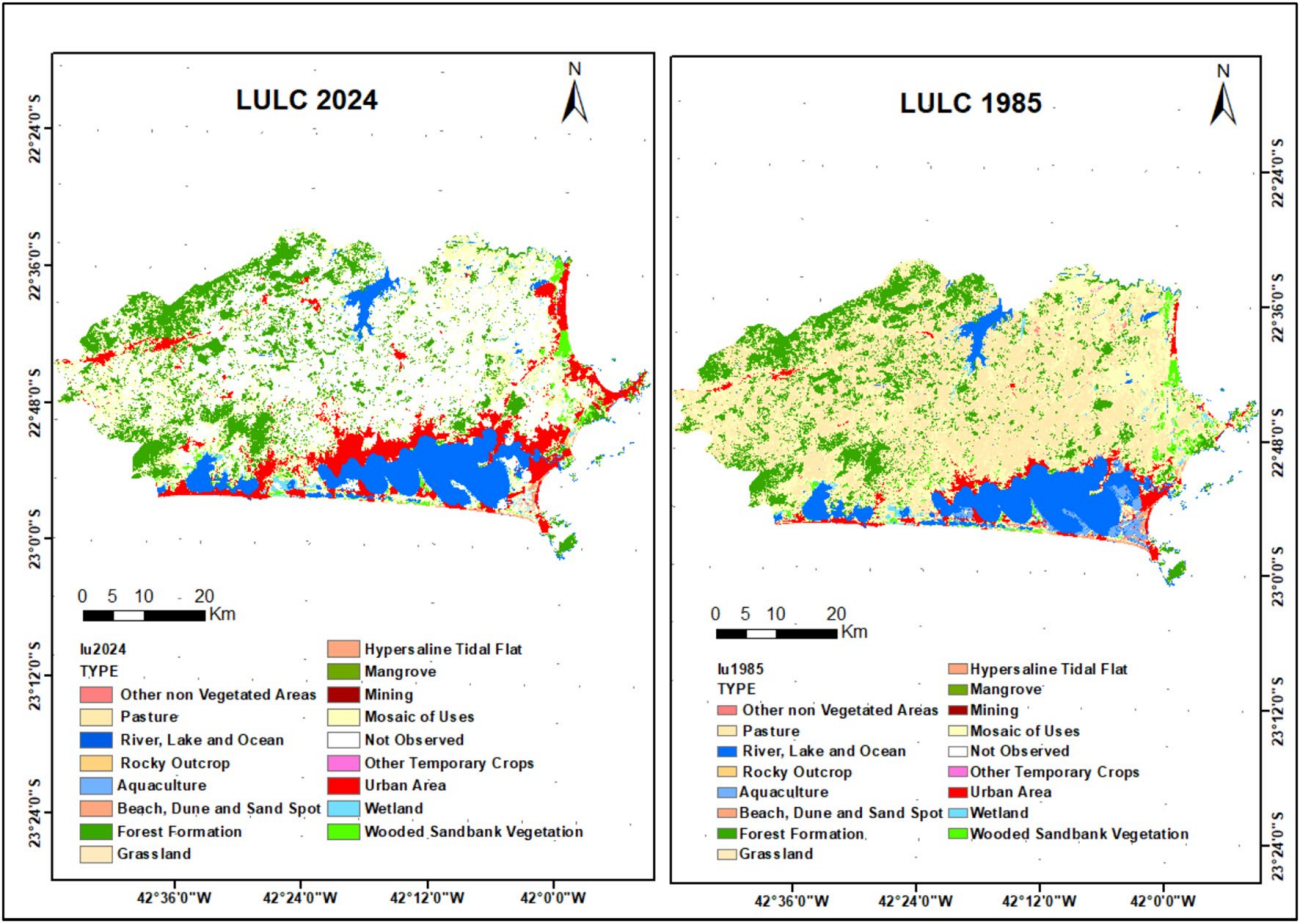
Landuse/Landcover Changes between 1985 and 2024. Map created using ArcGIS version 10.8 (https://www.esri.com/en-us/arcgis/products/arcgis-desktop/resources) with data from MapBiomas Project (https://mapbiomas.org).

Between 1985 and 2024, the study area experienced substantial shifts in land cover composition (Fig. 5). Urban areas exhibited the most pronounced expansion (Table 3 and 4), increasing by 199.42% (203.50 km^2^). Pastureland also expanded significantly, growing by 14.74% (167.61 km^2^), and remained the dominant land use category throughout the period. Mining activities increased dramatically by 6147.73% (from 0.04 km^2^ to 2.47 km^2^), albeit from a very small initial extent, while Other Temporary Crops expanded notably by 445.01%. Mangrove areas increased by 53.13% (+0.34 km^2^), suggesting positive restoration or conservation outcomes, and water bodies (River, Lake and Ocean) expanded by 7.38% (+23.26 km^2^). New land use categories emerged during the study period, including Sugar cane (0.64 km^2^ in 2024) and Forest Plantation (0.01 km^2^ in 2024).

**Table 4:** Consolidated land use & land cover changes (km^2^) 1985–2024.

**ID**	**LULC Classes**	**1985 (km**^**2**^**)**	**1995** **(km**^**2**^**)**	**2005** **(km**^**2**^**)**	**2015** **(km**^**2**^**)**	**2024** **(km**^**2**^**)**	**Net change** **(%)**	**Net change** **(km**^**2**^**)**
3	Forest formation	555.63	484.60	497.95	517.05	548.61	-1.26	-7.02
5	Mangrove	0.63	0.76	0.88	0.97	0.97	53.13	0.34
9	Forest plantation	0.00	0.00	0.00	0.01	0.01	100.00	0.01
11	Wetland	36.52	31.09	43.82	40.34	31.67	-13.30	-4.86
12	Grassland	0.46	0.44	0.43	0.44	0.25	-44.81	-0.21
15	Pasture	1137.16	1303.61	1384.17	1453.34	1304.77	14.74	167.61
20	Sugar cane	0.00	0.00	0.00	0.00	0.64	100.00	0.64
21	Mosaic of uses	1017.59	864.90	710.62	599.09	668.10	-34.35	-349.49
23	Beach, dune and sand spot	13.05	12.81	10.30	11.84	11.26	-13.73	-1.79
24	Urban area	102.05	166.33	214.70	254.15	305.55	199.42	203.50
25	Other non Vegetated Areas	15.54	14.75	–	22.01	18.84	21.21	3.30
29	Rocky outcrop	0.12	0.03	0.00	0.01	0.01	-88.37	-0.10
30	Mining	0.04	0.31	19.86	1.08	2.47	6147.73	2.43
31	Aquaculture	49.79	46.12	37.30	20.60	18.23	-63.39	-31.56
32	Hypersaline tidal flat	0.09	0.06	0.04	0.00	0.01	-90.91	-0.08
33	River, LAKE AND OCEAn	315.18	325.95	332.32	329.79	338.44	7.38	23.26
39	Soybean	0.00	0.00	0.00	0.05	0.00	0.00	0.00
41	Other temporary crops	0.55	5.79	1.03	5.58	2.98	445.01	2.43
49	Wooded sandbank vegetation	51.11	37.96	41.40	39.16	42.69	-16.47	-8.42

Conversely, several land cover classes experienced notable declines. Mosaic of Uses decreased by 34.35% (−349.49 km^2^), representing the largest absolute reduction. Aquaculture declined substantially by 63.39% (−31.56 km^2^), and Grassland diminished by 44.81%. Rocky Outcrop areas were reduced by 88.37%, while Wetlands decreased by 13.30% (−4.86 km^2^). Forest Formation showed a modest net decline of 1.26% (−7.02 km^2^), though it recovered somewhat after reaching its lowest extent in 1995. Other categories, such as Beach, Dune and Sand Spot and Wooded Sandbank Vegetation, also registered reductions of 13.73% and 16.47%, respectively.

#### Land use transition analysis and degradation linkages

To establish empirical relationships between land-use change and observed degradation patterns, a qualitative assessment was conducted focusing on land cover transitions that occurred between 1985 and 2024 within areas classified as unstable in the final degradation map. This analysis aimed to identify which land use transitions appeared most frequently associated with degradation hotspots. The assessment revealed that unstable areas were often associated with specific land use transition pathways. The conversion from Mosaic of Uses to Pasture was frequently observed in the central portion of the study area, particularly in municipalities such as Rio Bonito and São Vicente de Paula. These areas exhibited visible sheet erosion features, suggesting that pasture establishment following heterogeneous small-scale farming systems may involve land clearing practices that increase erosion vulnerability when soil conservation measures are absent. Another notable transition pathway was the conversion from Forest Formation to Mosaic of Uses, which was frequently observed in steeper terrain within Silva Jardim and Morro Grande. Forest clearing for mixed cropping systems appeared to increase erosion susceptibility, particularly during the initial clearing phase when soil remains exposed. The expansion of urban and peri-urban areas, often at the expense of former pasturelands, was also associated with degradation features. Soil exposure during construction activities and alterations to natural drainage patterns contributed to both sheet erosion and localized rill erosion along expanding urban peripheries. In contrast, areas that remained under continuous vegetation cover—particularly forest formations—showed limited evidence of active erosion, suggesting that stable land cover plays a protective role against degradation processes. Similarly, areas where natural regeneration of forest vegetation occurred appeared to exhibit greater stability, supporting the potential effectiveness of secondary forest regrowth as a restoration strategy.

These qualitative observations suggest that land use transitions—particularly those involving the removal of natural vegetation or the simplification of agricultural mosaics—may create conditions favorable to erosion processes. This finding points toward the importance of timing conservation interventions to coincide with periods of land use change, when technical assistance and soil conservation planning can be most effectively implemented.

## Discussion

### Discussion of land degradation patterns

The finding that stable land conditions dominate the study area (68.4%) aligns with national-scale analyses of Brazilian land cover, which show that despite intense historical pressure, significant portions of the landscape persist as stable natural vegetation or managed agricultural land^13^. The most significant stable classes—Unmanaged Areas with Agriculture and Forest Potential, collectively represent nearly half (48.7%) of the total landscape and constitute the region’s primary reservoir of productive and ecological capital. However, their classification as “unmanaged” or with specific “potential” indicates a latent vulnerability. These lands represent the very frontiers most susceptible to future conversion, a transition strongly associated with the initiation of soil degradation processes globally^14^ and identified as a primary driver of ecosystem change worldwide^1^. The high proportion of stable area, therefore, should be interpreted not as a lack of threat but as a critical window for implementing preventive land-use planning and sustainable management practices to avert future degradation.

Conversely, the mapped unstable areas, though covering a smaller portion of the landscape (7.8%), reveal clear and concerning spatial patterns that are critical for intervention. The predominance of sheet erosion—accounting for the majority (7.04%) of all unstable area—is consistent with global models identifying it as the most extensive form of soil loss by water^2,15^ and a major contributor to global land degradation^1^. The spatial correlation of these erosion hotspots with “agricultural frontiers” and interfaces between managed land and natural vegetation underscores a primary driver: land-use transition. This pattern aligns with the classic syndrome of land change where frontier expansion triggers soil degradation^14^. The resulting soil degradation is a direct consequence of anthropogenic pressure, occurring where natural systems are being modified or where agricultural practices may be exceeding the land’s carrying capacity. Similar intense anthropogenic pressures, notably urbanization and land-use change, are documented as critical factors altering both terrestrial and coastal ecosystems in southeastern Brazil^13,16^. The concentration of unstable areas in central and peripheral zones highlights specific localities where current land-use systems warrant urgent review. Targeted soil conservation measures, aligned with and reinforcing instruments such as Brazil’s Forest Code^17^, are most needed in these priority intervention zones.

### Discussion of conservation priorities and management implications

The priority classification provides a strategic, multi-tiered framework for directing conservation resources. The finding that over half of the landscape (51.69%) is classified as Stable Medium Priority is significant. This vast area represents lands that are currently stable but possess inherent or contextual vulnerabilities, aligning with the concept of “potential degradation” risk areas that require proactive management to maintain ecosystem services^4^. Investing in sustainable agricultural practices, agroforestry systems, and compliance with environmental legislation within these zones is a cost-effective strategy to prevent their transition into future degradation hotspots, an approach advocated for in preventative conservation planning^18^. In the Brazilian context, such preventive measures are central to the implementation of the Forest Code, which aims to protect areas of permanent preservation (APPs) and legal reserves to maintain ecological stability on private lands^19^^,^^17^.

Conversely, the Unstable High Priority areas (4.64% of the total), though smaller in extent, demand immediate and targeted rehabilitation efforts. The concentration of these critical zones at land-use interfaces underscores that degradation is a direct symptom of unsustainable transitions, a pattern well-documented in other global contexts^14^. The application of the UNEP/PAP/RAC framework in this humid tropical region successfully identified these hotspots, demonstrating its utility beyond the Mediterranean and semi-arid contexts for which it was originally developed (e.g.,^5,7^. The methodology’s structured integration of biophysical and socio-economic criteria offers a replicable model for translating complex degradation assessments into actionable intervention classes, which is a critical step for operationalizing land degradation assessments^20^.

This dual-focused strategy urgent rehabilitation of high-priority unstable areas (addressing existing degradation) coupled with the safeguarding of medium-priority stable lands (avoiding future degradation) is essential for achieving LDN targets, which require balancing “losses” to degradation against “gains” from improvement (UNCCD GPG v2, Section 1.4). The resulting priority map directly translates a multi-criteria assessment into a practical blueprint for environmental agencies, enabling efficient, spatially explicit allocation of resources. Such prioritization is fundamental for operationalizing LDN and aligning local actions with national LDN targets and SDG 15.3, as it moves beyond merely calculating the proportion of degraded land to informing where and how to act^21^. By emphasizing that preventive measures in stable areas are as crucial as rehabilitation in degraded ones, this study supports an integrated land management approach aimed at achieving a land degradation-neutral world.

### Discussion of LULC dynamics and degradation linkages

The observed land-use and land-cover (LULC) transitions in the study area from 1985 to 2024 align closely with broader regional dynamics documented in the Atlantic Forest biome and Rio de Janeiro state, where key drivers include agricultural expansion, urbanization, deforestation, and policy-induced recovery. Urban areas expanded dramatically by 203.50 km^2^ (199.42%), consistent with patterns of metropolitan growth and peri-urbanization across the state, driven by population influx and economic development^22^. This expansion often converts agricultural mosaics into built-up land, increasing pressure on surrounding rural landscapes. Concurrently, pasture expanded by 14.74% (167.61 km^2^) while Mosaic of Uses declined by 34.35% (349.49 km^2^), indicating a process of agricultural intensification and landscape simplification. This widespread trend in southeastern Brazil is fueled by demand for livestock products, where heterogeneous farming systems transition to more homogeneous pasturelands, often operating below their productive potential^17^.​ Forest Formation showed a modest net loss of 1.26% (7.02 km^2^) but exhibited recovery after 1995. This pattern mirrors the slow but positive stabilization and regrowth observed in the Atlantic Forest biome, driven by strengthened environmental legislation such as Brazil’s Forest Code and reduced deforestation^23^,^12^.

The qualitative transition analysis presented in Section "Land Use Transition Analysis and Degradation Linkages" provides insights into the relationship between land cover change and observed degradation hotspots. The conversion from Mosaic of Uses to Pasture emerged as a transition pathway frequently associated with unstable areas, particularly in the central portion of the study area. This finding aligns with the understanding that the simplification of heterogeneous agricultural systems into extensive pastureland—often without accompanying soil conservation practices—can increase erosion vulnerability. The sheet erosion features observed in these areas suggest that the process of land conversion itself, rather than the eventual land use alone, may create conditions favorable to soil loss. Similarly, the conversion from Forest Formation to Mosaic of Uses, observed in steeper terrain, highlights the erosion risks associated with forest clearing on vulnerable slopes. This pattern is consistent with global findings that deforestation on sloping lands accelerates runoff and soil loss, particularly during the initial post-clearing period when soil remains exposed^2^.

The expansion of urban areas at the expense of pasturelands was also associated with degradation features, particularly sheet and localized rill erosion. Soil exposure during construction, compaction from heavy machinery, and alterations to natural drainage patterns collectively contribute to increased erosion risk in peri-urban environments^16^. Notably, areas that remained under continuous forest cover or underwent natural regeneration appeared to exhibit greater stability, supporting the protective role of vegetation in maintaining soil integrity. This observation reinforces the importance of forest conservation and restoration as strategies for erosion control.

The finding that degradation hotspots are frequently associated with land use transitions—rather than stable land use persistence—has important policy implications. Interventions aimed at preventing degradation should be strategically timed to coincide with periods of land use change, when technical assistance, conservation planning, and soil conservation measures can be most effectively implemented. This approach aligns with the concept of "transition-driven degradation"^14^, where the process of land conversion creates windows of vulnerability that require targeted intervention. Divergent coastal ecosystem trends were observed: mangrove areas expanded while wetlands contracted. This highlights differential pressures from coastal development and aquaculture versus inland drainage for agriculture. Mangroves appear to have benefited from protective policies and natural expansion,studies in the Rio de Janeiro coastline document mangroves encroaching landward and expanding over salt marshes, often in response to local geomorphological shifts, sedimentation patterns, and relative sea-level rise^16^^,^^24^.

Finally, the emergence of sugarcane and the sharp rise in Other Temporary Crops signal nascent agricultural shifts tied to market demands and climate-adapted cropping in southeast Brazil, including Rio de Janeiro, necessitating ongoing monitoring (Maria^25^^,^^13^.

### Limitations and implications for management

This study provides a robust spatial assessment of land degradation and conservation priorities. However, certain limitations should be acknowledged to contextualize the findings.

#### Validation limitations

Systematic field-based ground-truthing was not conducted following the completion of the classification and mapping phases. To mitigate this limitation, the classification methodology incorporated several quality assurance measures: (1) iterative refinement of training samples using very-high-resolution (<1 m) Airbus and Esri basemaps; (2) careful visual interpretation and iterative refinement of training samples to ensure spectral discrimination between classes; and (3) strict adherence to the established UNEP-PAP/RAC framework, which has been validated in analogous environmental contexts. Nonetheless, future studies should prioritize systematic ground-truthing and independent accuracy assessment to further validate the classification, particularly for rill erosion subtypes which remain challenging to discriminate at medium resolution.

#### Prioritization framework limitations

The conservation prioritization, while based on a structured multi-criteria framework informed by expert judgment, has certain constraints. Socio-economic variables G (rural exodus), H (land tenure), and I (other aggravating socio-economic factors) were assigned the lowest impact score (1) for all land units because no field survey or questionnaire was conducted. Future applications of this framework would benefit from participatory engagement with local farmers, landowners, and community representatives to capture nuanced socio-economic values, land tenure complexities, and local ecological knowledge, thereby refining the scores for these variables.

#### Data accessibility limitations

Following the completion of the analysis, access to the geospatial datasets and computational infrastructure was no longer available. Consequently, certain quantitative validations and statistical analyses that would ideally accompany a study of this nature could not be performed. The authors have addressed this by presenting findings qualitatively where precise statistics could not be verified, and by emphasizing the methodological transparency of the approach.

#### Implications for management

Despite these limitations, the findings offer clear and actionable implications for land management in the State of Rio de Janeiro. The spatially explicit priority map (Fig. 4) serves as a direct decision-support blueprint for environmental agencies at state and municipal levels. It enables the efficient direction of technical assistance, conservation incentives (e.g., payments for ecosystem services), and regulatory enforcement towards the most critical intervention hotspots, particularly the Unstable High Priority areas identified in municipalities such as Rio Bonito and Boa Esperança.

The results advocate for an integrated, two-pronged management strategy essential for achieving land degradation neutrality. This strategy must couple the urgent rehabilitation of high-priority unstable areas with robust policies and incentives that safeguard the extensive Stable Medium Priority lands from future degradation. Investing in sustainable agricultural practices, agroforestry, and adherence to the Forest Code within these stable-yet-vulnerable zones represents a cost-effective, preventative approach to conservation.

By implementing this dual focus, policymakers and land managers can work towards ensuring the long-term ecological functionality and economic productivity of this environmentally significant region.

## Recommendations

Based on the prioritization of land degradation hotspots in southeastern Brazil, we propose targeted interventions tailored to the socio-environmental context of the Rio de Janeiro study area.

Firstly, for the unstable Areas: these areas, encompassing High Priority (140.17 km^2^) and Medium Priority (77.97 km^2^) zones, are characterized by active sheet and rill erosion, often at agricultural frontiers and land-use interfaces. Curative and protective interventions include:

- Soil Conservation Infrastructure: implement contour bunds, terraces, and vegetative barriers in agricultural zones experiencing dominant sheet erosion to reduce runoff velocity and soil loss.
- Ecological Restoration: reforest critical high-priority zones using native Atlantic Forest species to stabilize degraded slopes and restore hydrological function, particularly in areas adjacent to remaining forest fragments.
- Governance and Enforcement: strengthen the enforcement of the Brazilian Forest Code, ensuring the protection and restoration of Permanent Preservation Areas (APPs) and Legal Reserves within agricultural properties.
- Sustainable Land Management Support: provide technical assistance and incentives to farmers in erosion-prone areas for adopting soil conservation practices and integrated crop-livestock-forestry systems.

Secondly, for the At-Risk Stable Areas: measures for these currently stable but vulnerable lands, primarily classified as Medium Priority (1560.80 km^2^) focus on maintaining stability and preventing future degradation:

- Sustainable Agricultural Intensification: Promote conservation agriculture, agroforestry, and soil cover maintenance in unmanaged areas with agricultural potential to prevent their conversion into future erosion hotspots.
- Land-Use Zoning and Planning: integrate degradation risk maps into municipal and regional land-use planning (Zoneamento Ecológico-Econômico - ZEE) to guide sustainable development away from high-risk zones and protect areas with high conservation value.
- Payment for Ecosystem Services (PES): develop and implement PES schemes to incentivize landowners in stable medium-priority areas to maintain forest cover, adopt sustainable practices, and avoid land conversion.

To enhance feasibility, these measures should be integrated into existing state and municipal environmental policies and supported by funding mechanisms such as environmental compensation and the Brazilian Fund for Biodiversity (FUNBIO). Future efforts must prioritize participatory validation of the mapped degradation zones with local stakeholders and ground-truthing of erosion processes. By situating the PAP/RAC framework within Brazil’s robust environmental governance structure, this study provides a replicable, spatially explicit model for guiding soil conservation, ecosystem restoration, and sustainable land-use planning in tropical coastal regions.

## Conclusion

This study successfully applied an integrated remote sensing and GIS-based UNEP-PAP/RAC framework to assess land degradation risk and prioritize conservation needs in a humid tropical coastal region of southeastern Brazil. The findings reveal a landscape dominated by stable yet vulnerable lands, with significant areas classified as medium priority for preventive conservation. Critically, active degradation hotspots were clearly identified, primarily linked to agricultural frontiers and land-use transitions, underscoring the role of anthropogenic pressure in driving soil erosion. The resulting spatially explicit priority maps provide a science-based, actionable tool for land managers and policymakers, enabling targeted interventions to rehabilitate high-risk areas while safeguarding stable zones from future degradation. Along with the targeted interventions recommended, this approach supports the strategic pursuit of land degradation neutrality and sustainable land management in one of Brazil’s most dynamic and environmentally significant regions.

## Data Availability

Data fully available upon request

## References

[1] IPBES *The IPBES assessment report on land degradation and restoration*. Secretariat of the Intergovernmental Science-Policy Platform on Biodiversity and Ecosystem Services. 10.5281/zenodo.3237392 (2018).

[2] Borrelli, P. et al. An assessment of the global impact of 21st century land use change on soil erosion. *Nat. Commun.* **8**(1), 1–13. 10.1038/s41467-017-02142-7 (2017). 29222506 10.1038/s41467-017-02142-7 PMC5722879

[3] Priority Actions Program Regional Activity Centre (PAP/RAC) *Guidelines for mapping and measurement of rainfall-induced erosion processes in the Mediterranean coastal areas*. Split, Croatia. (1997).

[4] Visser, S., Keesstra, S., Maas, G. & de Cleen, M. Soil as a basis to create enabling conditions for transitions towards sustainable land management as a key to achieve the SDGs by 2030. *Sustainability* **11**(23), 1–19. 10.3390/su11236792 (2019).

[5] Lhoussaine, E. M. et al. A GIS-based modified PAP/RAC model and Caesium-137 approach for water erosion assessment in the Raouz catchment, Morocco. *Environ. Res.* **251**(1), 118–460. 10.1016/j.envres.2024.118460 (2024). 10.1016/j.envres.2024.118460 38387493

[6] Mesrar, H. et al. Modélisation de l’érosion hydrique et des facteurs causaux: cas de l’Oued Sahla, rif central, *Maroc*. *Z. Geomorphol.* **59**(3), 495–514 (2015).

[7] Sadiki, A., Mesrar, H. & Faleh, A. Modélisation et cartographie des risques de l’érosion hydrique: cas du bassin versant de l’Oued Larbaa, Maroc. *Pap. Geogr.* **55–56**, 179–188 (2012).

[8] Tahouri, J. et al. Using a modified PAP/RAC model and GIS for mapping water erosion and causal risk factors: Case study of the Asfalou watershed, Morocco. *Int. Soil Water Conserv. Res.* **10**, 254–272 (2022).

[9] AlAbed, M., Saboya, M., Santos, C., Vargas, R. & Ferreira Dias, F. Land degradation assessment in the Angra dos Reis (Brazil) using remote sensing and GIS. *J. Agric. Sci. Technol.* **2&3**(1), 40–56. 10.36108/jast/5202.320.0130 (2025).

[10] United Nations Environment Programme (UNEP)/Mediterranean Action Plan (MAP)/ Priority Actions Programme (PAP). *Guidelines for erosion and desertification control management with particular reference to Mediterranean coastal areas*. https://iczmplatform.org/storage/documents/Vn1Imo6Q5bY3ozfyUJy0B2tnXdwHII5K8PlKZdlo.pdf (2000).

[11] United Nations Environment Programme (UNEP)/ Mediterranean Action Plan (MAP)/ Priority Actions Programme (PAP). *Improving coastal land degradation monitoring in Lebanon and Syria: Country report Syria*. https://wedocs.unep.org/bitstream/handle/20.500.11822/1859/syria.pdf

[12] MapBiomas. https://Brazil.mapbiomas.org/en/2025/08/13/Brazil-quatro-decadas-de-transformacao-na-cobertura-e-uso-da-terra-revelam-desafios-e-oportunidades/ (2025).

[13] Souza, C. M. Z. et al. Reconstructing three decades of land use and land cover changes in Brazilian biomes with Landsat archive and Earth Engine. *Remote Sens.* **12**(17), 1–27. 10.3390/rs12172735 (2020).

[14] Lambin, E. F. & Meyfroidt, P. Global land use change, economic globalization, and the looming land scarcity. *Proc. Natl. Acad. Sci. U. S. A.* **108**(9), 3465–3472 (2011). 21321211 10.1073/pnas.1100480108 PMC3048112

[15] Panagos, P. et al. Cost of agricultural productivity loss due to soil erosion in the European Union: From direct cost evaluation approaches to the use of macroeconomic models. *Land Degrad. Dev.* **29**(3), 471–484 (2018).

[16] Santos, C. A. et al. Spatio-temporal evolution of environmental dynamics in Guaratiba State Biological Reserve and its surroundings, Rio de Janeiro, Brazil. *Caminh. Geogr.* **23**(90), 120–138. 10.14393/RCG239061036 (2022).

[17] Strassburg, B. N. et al. When enough should be enough: Improving the use of current agricultural lands could meet production demands and spare natural habitats in Brazil. *Glob. Environ. Change* **8**, 84–97. 10.1016/j.gloenvcha.2014.06.001 (2014).

[18] Nkonya E, Mirzabaev A and von Braun J (Eds.) *Economics of land degradation and improvement – a global assessment for sustainable development*. Springer International Publishing. 10.1007/978-3-319-19168-3 (2026).

[19] Soares-Filho, B. et al. Cracking Brazil’s forest code. *Science* **344**(6182), 363–364. 10.1126/science.1246663 (2014). 24763575 10.1126/science.1246663

[20] Kust, G., Andreeva, O. & Cowie, A. Land degradation neutrality: Concept development, practical applications and assessment. *J. Environ. Manage.* **195**(Pt 1), 16–24. 10.1016/j.jenvman.2016.10.043 (2017). 27825772 10.1016/j.jenvman.2016.10.043

[21] Sims, N. C. et al. *Good Practice Guidance. SDG Indicator 15.3.1, Proportion of Land That Is Degraded over Total Land Area. Version 2.0* (United Nations Convention to Combat Desertification, 2021).

[22] Pereira, R. H. M. Future accessibility impacts of transport policy scenarios: Equity and sensitivity to travel time thresholds for bus rapid transit expansion in Rio de Janeiro. *J. Transp. Geogr.* **74**, 321–332. 10.1016/j.jtrangeo.2018.12.005 (2019).

[23] Rezende, C. L. et al. From hotspot to hopespot: an opportunity for the Brazilian Atlantic Forest. *Perspect. Ecol. Conserv.* **16**(4), 208–214. 10.1016/j.pecon.2018.10.002 (2018).

[24] Costa Santos, C. S. et al. Relative sea level rise effects at the Marambaia Barrier Island and Guaratiba Mangrove: Sepetiba Bay (SE Brazil). *J. Sediment. Environ.* **4**(3), 249–62. 10.12957/jse.2019.44397 (2019).

[25] Maria Guimarães A new chance for the Atlantic Forest*. Revista Pesquisa FAPESP*. Issue # 237. https://revistapesquisa.fapesp.br/en/a-new-chance-for-the-atlantic-forest/ (2025).

